# Wavelet analysis of laser Doppler microcirculatory signals: Current applications and limitations

**DOI:** 10.3389/fphys.2022.1076445

**Published:** 2023-01-20

**Authors:** Lana Kralj, Helena Lenasi

**Affiliations:** ^1^ Institute of Physiology, Faculty of Medicine, University of Ljubljana, Ljubljana, Slovenia

**Keywords:** laser Doppler flowmetry, wavelet analysis, spectral analysis, frequency band, microcirculation, endothelial function

## Abstract

Laser Doppler flowmetry (LDF) has long been considered a gold standard for non-invasive assessment of skin microvascular function. Due to the laser Doppler (LD) microcirculatory signal’s complex biological and physiological context, using spectral analysis is advisable to extract as many of the signal’s properties as feasible. Spectral analysis can be performed using either a classical Fourier transform (FT) technique, which has the disadvantage of not being able to localize a signal in time, or wavelet analysis (WA), which provides both the time and frequency localization of the inspected signal. So far, WA of LD microcirculatory signals has revealed five characteristic frequency intervals, ranging from 0.005 to 2 Hz, each of which being related to a specific physiological influence modulating skin microcirculatory response, providing for a more thorough analysis of the signals measured in healthy and diseased individuals. Even though WA is a valuable tool for analyzing and evaluating LDF-measured microcirculatory signals, limitations remain, resulting in a lack of analytical standardization. As a more accurate assessment of human skin microcirculation may better enhance the prognosis of diseases marked by microvascular dysfunction, searching for improvements to the WA method is crucial from the clinical point of view. Accordingly, we have summarized and discussed WA application and its limitations when evaluating LD microcirculatory signals, and presented insight into possible future improvements. We adopted a novel strategy when presenting the findings of recent studies using WA by focusing on frequency intervals to contrast the findings of the various studies undertaken thus far and highlight their disparities.

## 1 Introduction

Skin microcirculation^1^ is a network of blood vessels less than 150 µm in diameter, consisting of arterioles, capillaries, and venules (Rizzoni et al., 2011; Strain and Paldánius, 2018). Due to its thermoregulatory function, this network plays a major role in maintaining body homeostasis (Holowatz et al., 2008; Lenasi, 2011; Treu et al., 2017; Strain and Paldánius, 2018). Various mechanisms, including neural and humoral ones, participate in controlling microvascular reactivity by adapting vessel diameter according to the body’s current thermoregulatory needs (Johnson and Kellogg, 2010). The interactions between these mechanisms are complex (Cracowski and Roustit, 2020) and differ according to measuring site (Lenasi, 2016; Potočnik and Lenasi, 2016), resulting in considerable skin blood flow (SkBF) variation and, accordingly, considerable variation in the measured signal. The neural control mechanisms regulating microvascular reactivity differ between the glabrous (non-hairy) and non-glabrous (hairy) skin sites. While the role of the sympathetic vasoconstrictor fibers, which innervate most parts of the skin, is well understood, the mechanisms contributing to vasodilation in non-glabrous skin, and believed to be mediated by distinct nerve fibers are far less elucidated; it has been suggested that sympathetic active vasodilatory nerve fibers with a neurotransmitter yet to be defined play a role (Lenasi, 2011). In addition, various factors affect the neurally mediated mechanisms, ultimately modulating the degree of cutaneous vasoconstriction and vasodilation (Charkoudian and Johnson, 2000; Aoki et al., 2003; Wong and Hollowed, 2016).

The role of the vascular endothelium is considered of great importance when addressing microcirculatory control mechanisms independent of neural and humoral factors (Lenasi, 2011). However, because the contribution of various endothelium-derived vasodilators depends on the skin site as well as on the microvascular size (Félétou and Vanhoutte, 2009), the exact contribution of the endothelial component to the regulation of vascular tone is difficult to define.

Furthermore, vasomotion, the spontaneous, autonomous oscillation in a tone of blood vessel walls, independent of the heart, innervation, respiration, and endothelium (Lenasi, 2011) should also be considered when discussing microcirculatory control mechanisms. Currently, it is believed that vasomotion is associated with the intrinsic myogenic activity of the vascular smooth muscle cells (vSMC) (Silva et al., 2021); however, the exact mechanisms underlying this phenomenon require elucidation (Aalkjaer et al., 2011).

Dysfunctional microcirculation is related to several metabolic and cardiovascular diseases and conditions such as coronary artery disease (Ijzerman et al., 2003), diabetes mellitus (DM) type 1 (DM1) and type 2 (DM2) (Sorelli et al., 2019), hypercholesterolemia, arterial hypertension (AH), and obesity (Mahé et al., 2012; Gutterman et al., 2016). Furthermore, microvascular dysfunction may constitute the first sign of disease development and be present prior to clinical signs (Mizeva et al., 2018; Andreieva et al., 2021), making its evaluation an important step in disease risk stratification.

To gain optimal insight into the complex control mechanisms of skin microcirculation in normal and diseased states, various optical imaging techniques, coupled with different pharmacological or physical stimuli to challenge microvascular reactivity, are usually used (Mizeva et al., 2018). Laser Doppler flowmetry (LDF)^2^, which provides non-invasive, semi-quantitative, and real-time microcirculatory signal tracking (Mahé et al., 2012; Iredahl et al., 2015; Rodrigues et al., 2019), is still considered the gold standard (Lenasi, 2011).

Because the biological and physical context of the laser Doppler (LD) signal is complex, the method for analyzing it should be chosen carefully. It has been established that using spectral analysis allows for greater information extraction than simply analyzing flow values over time (Bagno and Martini, 2016). Spectral analysis decomposes the LD signal into typical spectra consisting of frequency intervals related to various physiological influences modulating microcirculation, allowing a more thorough analysis of the LD signals.

Spectral analysis can be performed using either a classical Fourier transform (FT) technique, which has the disadvantage of not being able to localize a signal in time, or wavelet analysis (WA), which provides for both time and frequency localization of the signal inspected (Stefanovska et al., 1999). Given the increasing acceptance of WA as a suitable technique for evaluating microcirculatory signals, there is a need for critical review of this technique and investigation of possible improvements. In our article, we summarize and discuss the current applications and limitations of WA in evaluating the microcirculatory signal measured with LDF. In addition, we adopt a novel strategy to present the results of recent WA studies by focusing on frequency intervals rather than diseases to best compare and contrast the results of the various studies performed to date and to highlight the differences between the various studies. In our opinion, this approach uses practical examples to highlight the main advantages and disadvantages of WA, the resolution of which would undoubtedly result in a more accurate assessment of skin microvascular function and potentially contribute to earlier detection of microvascular dysfunction at the clinics.

## 2 Wavelet analysis: Physical principles

FT is a standard spectral decomposition tool that, when applied to a signal of interest, uses sine and cosine as basis functions to decompose a signal into its frequency components. FT first translates a function from the time domain to the frequency domain. The Fourier coefficients of the transformed function represent the contribution of each sine and cosine function at each frequency (Graps, 1995). In practice, the Fast Fourier Transform (FFT) algorithm, which effectively implements the FT by providing a fast decomposition of a function into sine and cosine series, is considered the onset spectral analysis technique to which other techniques are compared (Herff and Krusienski, 2018).

**FIGURE 1:**
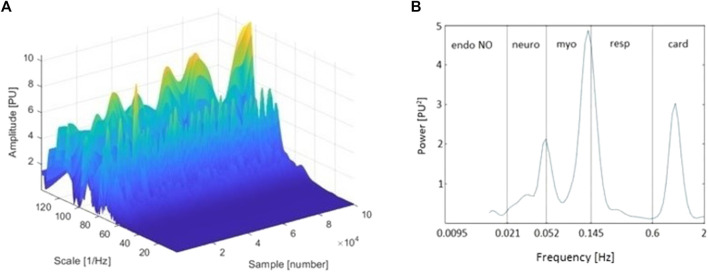
**(A)** Three-dimensional wavelet transform (WT) of the laser Doppler (LD) signal of a representative tracing (baseline period, finger pulp); and **(B)** Time-averaged wavelet power spectrum of the same signal and frequency intervals corresponding to endothelial nitric oxide (NO)-dependent (endo NO), neurogenic (neuro), myogenic (myo), respiratory (resp), and cardiac (card) physiological influences. The sample axis on the three-dimensional spectrum represents the sampling points of the signal. Thereby, it can also be observed as a time axis **(A)**.

Even though the FT approach provides good localization in terms of a signal’s frequency domain, it does not provide for the time localization of the observed frequencies. Considering that the oscillatory processes involved in the regulation of microcirculation, reflecting various physiological influences and ranging from 0.005 to 2 Hz, presumably change with time rather than frequency (Bernjak and Stefanovska, 2007; Ažman-Juvan et al., 2008; Sun et al., 2012), the inability to track variations of characteristic frequency with time presents a serious drawback. Although the short-time FT (STFT) partially solves this problem by using a fixed-length window function (usually a rectangle) to provide a time-frequency representation of a given signal (multiplying the original function by a finite-rectangle window function results in a zero signal everywhere except within the window of interest), it results in a fixed resolution that provides frequency distributions rather than peaks; that is, it provides information about which frequency bands exist at given time intervals, rather than about which frequencies exist at given time instances. This may not be sufficient to reliably reconstruct the time-frequency content of the complex microcirculatory signals, whose frequency content varies greatly over time. To overcome this limitation, the wavelet transform (WT) can be used. The main advantage of the WA approach over FT is its adjustable window function, which allows for the analysis of signals into different frequencies at different resolutions. This feature makes the WA approach suitable for the analysis of both high- and low-frequency biological LD signals (Bernjak and Stefanovska, 2007) and produces a much finer time-frequency representation, where the characteristic frequencies of the signal are represented as peaks in the 3D graphical representations with time-varying amplitude and position in frequency (Ažman-Juvan et al., 2008), as shown on the reconstructed 3D wavelet spectra in Figure 1A. Another important advantage of WA is its ability to detect discontinuities, edges, and/or signal overlaps, which makes it suitable for reconstruction of finite, non-periodic, transient, and non-stationary biological signals (Wahab and O’Haver, 2020). To evaluate microcirculatory function, physical or chemical provocations have typically been used to challenge its reactivity, inducing a time-localized transient phenomena that cannot be adequately assessed with conventional tools such as the FFT (Herff and Krusienski, 2018). To this end, WA provides an excellent and unique way to reliably track transient phenomena and is therefore generally considered more suitable than FT.

### 2.1 Mathematical definition

Unlike FT, WT does not use a single set of basis functions. Instead, an infinite set of possible basis functions, which comprise wavelet function families, is used (Graps, 1995). In continuous^3^ WA, a family of wavelet functions *ψ*_*τ*,*s*_, generated from a mother wavelet *ψ*(*t*) is defined as:

$$\psi_{\tau ,s}=\frac{1}{\sqrt{s}}\psi \left(\frac{t-\tau }{s}\right).$$ (1)

In the equation above, the parameter *s* represents scale, i.e., it stretches and compresses the wavelet $\left(s=\frac{1}{frequency},s\in R\backslash \left\{0\right\}\right)$ , and the parameter *τ* ∈ *R* represents the time-translation parameter. As a result, a wavelet can be observed as an adjustable window function that resolves low-frequency signal components using expanded wavelets (large values of scales, low time, and good frequency resolution) and higher frequencies using narrow wavelets (small values of scales, good time, and low-frequency resolution) (Silva et al., 2021).

Generally, the wavelet function *ψ*(*t*) must have a zero area:

$$\int_{-\infty }^{\infty }\psi \left(t\right)dt=0,$$ (2)

and its square should integrate to unity (Tankanag and Chemeris, 2008; Wahab and O’Haver, 2020):

$$\int_{-\infty }^{\infty }\psi^{2}\left(t\right)dt=1.$$ (3)

The continuous WT of a function *f*(*t*), which fulfills conditions (2) and (3), is defined by the following equation:

$$F\left(\tau ,s\right)=\frac{1}{\sqrt{s}}\int_{-\infty }^{\infty }f\left(t\right)\psi^{*}\left(\frac{t-\tau }{s}\right)dt.$$ (4)

It is important to note that choosing the mother wavelet is not straightforward. An appropriate function should be chosen to meet the purpose of the analysis. For studying the microcirculatory signal obtained by LDF, Morlet’s waveform family is commonly recommended (Stefanovska et al., 1999).

The Morlet wavelet, described by Eq. 5, is a complex function with a center frequency of *f*_0_ and a Gaussian envelope (described by the factor $e^{\left(-t^{2}/2\right)}$ ), and it demonstrates good localization in both time and frequency domains (Delvecchio, 2012):

$$\psi {\left(t\right)}_{\mathrm{Morlet}}=\frac{1}{\pi^{-1/4}}e^{\frac{-t^{2}}{2}}e^{i2\pi f_{0}t}.$$ (5)

### 2.2 Filtering

Finally, because filtering is widely used in the preprocessing of microcirculatory signals, the role of WA in this process should also be mentioned at this point. Namely, WT can be used to denoise the spectra of complex microcirculatory signals, such as signals obtained with photoplethysmography (PPG)^4^, which are often spoiled by Gaussian-like motion artifacts caused by the patient’s movements (Bhoi and Sarkar, 2012; Rodrigues et al., 2019; Liu et al., 2021). Although the FFT and conventional filters are also used for denoising (Herff and Krusienski, 2018; Rodrigues et al., 2019), the FFT has been shown to perform poorly on quasi-periodic data sets (Bhattacharya et al., 2001; Liu et al., 2021). Moreover, both conventional filters and the FFT can deform the microcirculatory signals and remove useful information (Huimin et al., 2012; Liu et al., 2021). The simplest WA-based filtering involves a few basic steps, including decomposing a signal using a (discrete) wavelet decomposition, filtering the signal using thresholds, and inverting the filtered signal back by performing inverse WT. In short, the general idea of this approach is to use WA to decompose the signal into details that, on a given scale, mainly contain the unwanted noise, which is then removed using thresholding without affecting the signal, as is often the case with other filtering techniques. For a more detailed discussion of selecting appropriate thresholds and other wavelet-based filtering methods, see for example Huimin et al. (2012).

### 2.3 Frequency intervals and physiological influences

Human skin microcirculatory signals demonstrate non-linear, periodical, oscillatory patterns, arising from various physiological influences. So far, spectral analyses of LD microcirculatory signals measured in healthy subjects have revealed five characteristic frequency intervals ranging from 0.005 Hz to 2 Hz (Häfner et al., 2007) (Figure 1B). It has been proposed that these intervals correspond to cardiac (0.6–2 Hz), respiratory (0.145–0.6 Hz), myogenic (0.052–0.145 Hz), neurogenic (0.021–0.052 Hz), and endothelial (0.005–0.021 Hz) physiological influences (Bagno and Martini, 2015). Considering the existence of different vasodilators, oscillations related to endothelial influence are occasionally observed within two frequency bands: the endothelial nitric oxide (NO)-dependent (0.0095–0.021 Hz) and the endothelial NO-independent one (0.005–0.0095 Hz) (Silva et al., 2021).

The oscillations related to cardiac, respiratory, and neurogenic motion appear to be influenced by centrally mediated mechanisms, whereas the oscillations with a frequency of around 0.1 Hz correspond to local mechanisms related to the intrinsic myogenic activity of vSMC. Finally, the oscillations with a frequency around 0.01 Hz or lower are associated with the influence of the vascular endothelium (Landsverk et al., 2007).

The primary purpose of performing WA on microcirculatory signals measured with LDF is to quantify the contribution of these frequency bands to the overall SkBF control. This is usually done by averaging reconstructed 3D wavelet spectra over time and calculating various WA parameters in the frequency bands of interest. Because WA frequency band parameters have been shown to be altered in disease (Tables 1, 2), this quantification may help to further elucidate the mechanisms of microvascular blood flow regulation and identify potential disruption of these mechanisms in diseased conditions.

**TABLE 1:** The impact of cardiac, respiratory, myogenic, neurogenic, and endothelial frequency bands determined by wavelet analysis.

Authors (year)	Subject	Provocation	Measurement site	Measurement duration	WT measure/WT family	Affected frequency bands
Mallette et al. (2017)	Passive heat stress in healthy individuals	Passive heating	Dorsal aspect of the forearm	20 min	Median power (MP)/Morlet WT	All bands *↑* besides endothelial NO-dependent
Mizeva et al. (2018)	Thermal testing in DM 1 and 2 patients	1. Cooling 2. Local heating	Glabrous skin of the foot	22 min	Spectral energy/Morlet WT	Endothelial *↓* Neurogenic *↓*
Jan et al. (2013)	Thermal testing in DM2 patients with peripheral neuropathy	Local heating	First metatarsal head of the right foot	50 min	Normalized amplitude (NA)/Morlet WT	Neurogenic *↓* Myogenic *↓* Endothelial *↓*
Jan et al. (2013)	Stress testing in DM2 patients with peripheral neuropathy	Local mechanical stress	First metatarsal head of the right foot	30 min	Normalized amplitude (NA)/Morlet WT	Myogenic *↓*
Silva et al. (2021)	Acute hyperglycemia	OGL, brachial artery occlusion	Forearm Finger pulp	25 min	Frequency interval activity/Morlet WT	Finger pulp: Neurogenic *↑* Cardiac *↓*
Landsverk et al. (2007)	General anesthesia	General anesthesia	Volar aspect of the lower forearm	22 min	Relative amplitude at frequency interval/Morlet WT	Respiratory *↑* Myogenic *↓* Neurogenic *↓* Endothelial *↓*
Andreieva et al. (2021)	Obesity	Brachial artery occlusion	Dorsum of the hand	10 min	Maximum amplitude at frequency interval (Amax)/Morlet WT	Endothelial *↓* Cardiac *↑*
Ažman-Juvan et al. (2008)	Acute myocardial infarction (AMI)	–	All extremities	30 min	Mean amplitude at frequency interval (MA)/Morlet WT	All bands *↓*
Rodrigues et al. (2020)	Lower limb massage	Lower limb effleurage massage applied in the proximal (p) and distal (d) direction	Plantar aspects of the second and first toes	25 min	Frequency interval activity/Morlet WT	Proximal: Cardiac *↑* (p,d) Respiratory *↑* (p,d) Myogenic *↑* (d)
Rossi et al. (2011)	Hypertension	WA performed on resting SkBf measurements	Right forearm	40 min	Absolute amplitude at frequency interval/Morlet WT	All bands *↑* besides myogenic *↓*

**TABLE 2:** Overview of quantitative results of studies presented in Table 1. NA-normalized amplitude, Amax-maximum amplitude, MA-mean amplitude. PU-perfusion units.

Authors (year)	Quantitative results
Mallette et al. (2017)	Cardiac: (0.529 ± 0.589 PU) vs*.* (0.858 ± 0.308 PU), respiratory: (0.283 ± 0.519 PU) vs*.* (0.789 ± 0.408 PU), myogenic: (0.482 ± 0.366 PU) vs*.* (0.976 ± 0.426 PU), neurogenic: (0.346 ± 0.181 PU) vs*.* (0.407 ± 0.111 PU), endothelial NO-independent: (0.173 ± 0.107 PU) vs*.* (0.264 ± 0.120 PU). The results (mean group spectral power ±standard error of the mean) are presented before vs. after exposing subjects to passive heating. Presented differences all exhibited statistical significance
Mizeva et al. (2018)	Numerical values for component analysis are not specified, the results are depicted in Mizeva et al. (2018)
Jan et al. (2013)	Numerical values for component analysis are not specified in case of performing thermal testing. The results are depicted in Jan et al. (2013)
Jan et al. (2013)	NA of myogenic frequency band in diabetics exposed to the mechanical stress test: (0.15 ± 0.032) vs*.* NA of myogenic frequency band in healthy controls exposed to the mechanical stress test: (0.36 ± 0.12). The results (mean group NA ± standard error) exhibited statistically significant difference
Silva et al. (2021)	Finger pulp: the sympathetic component increased between pre-load and post-load periods with glucose (group median 3.2 PU), whereas it decreased with water (group median 3.7 PU), a difference exhibiting statistical significance. The cardiac component decreased between pre-load and post-load periods with glucose (group median -1.1 PU), whereas it increased with water (group median 0.6 PU), a difference exhibiting statistical significance
Landsverk et al. (2007)	Numerical values for component analysis are not specified, the results are depicted in Landsverk et al. (2007)
Andreieva et al. (2021)	AmaxE reduced by 14.7% (group 1), 37.7% (group 2), 52.4% (group 3), 57.4% (group 4) when compared to healthy controls AmaxC reduced by 28.9% (group 1), 36.8% (group 2), 42.2% (group 3), 60.5% (group 4) when compared to healthy controls. Group 1: BMI (25–29.9 kg/m^2^), group 2 (30–34.9 kg/m^2^), group 3 (35–39.9 kg/m^2^), group 4 (≥40 kg/m^2^). Presented differences exhibited statistical significance
Ažman-Juvan et al. (2008)	Right arm: Cardiac MA 9.0 (4.5, 17.0) AMI vs*.* MA 26.9 (14.1, 49.8) controls, respiratory MA 8.7 (3.2, 17.3) AMI vs*.* MA 18.3 (12.0, 31.1) controls, myogenic MA 29.4 (9.3, 56.0) AMI vs*.* MA 62.6 (40.2, 113.2) controls, neurogenic MA 62.6 (20.7, 144.7) AMI vs*.* MA 107.3 (71.2, 185.8) controls, endothelial No-dependent MA 98.9 (35.1, 178.0) AMI vs*.* MA 165.2 (90.5, 300.9) controls, endothelial No-independent MA 108.0 (47, 254.2) AMI vs*.* 215.5 (130, 415.1) controls. Data are given as group median values with 25th and 75th percentiles in parentheses. Presented differences exhibited statistical significance. For the results provided from left arm, right leg and left leg, see Ažman-Juvan et al. (2008)
Rodrigues et al. (2020)	Numerical values for component analysis are not specified, the results are depicted in Rodrigues et al. (2020)
Rossi et al. (2011)	Numerical values for component analysis are not specified, the results are depicted in Rossi et al. (2011)

Most often, the absolute oscillation amplitude (Landsverk et al., 2007), the mean oscillation amplitude (Jan et al., 2013; Ažman-Juvan et al., 2008), the maximum oscillation amplitude (Andreieva et al., 2021), mean or median power (Bagno and Martini, 2015; Mallette et al., 2017), and spectral energy (Mizeva et al., 2018) in the frequency range of interest are used as WA parameters and expressed in perfusion units (PU).

In addition, it is possible to express a contribution of the particular frequency interval in relative measures. The approaches to calculating relative measures differ between authors. Some of these approaches include, but are not limited to, dividing the absolute oscillation amplitude in the frequency range of interest by the mean amplitude of the entire spectrum (relative amplitude) (Landsverk et al., 2007), dividing the mean amplitude of a particular frequency interval by that of the same frequency band of the baseline SkBF (normalized amplitude) (Jan et al., 2013), dividing the area under the curve of each component by the area under the curve of the entire spectrum (activity) (Rodrigues et al., 2020; Silva et al., 2021), or dividing the mean power of a particular frequency interval by the mean power of the entire spectrum (relative power) (Bagno and Martini, 2015).

## 3 Wavelet analysis: Physiological applications

Information on the applicability of WA in humans is rather scant in the available literature. So far, WA of LD signals has been performed to elucidate the role of microcirculation in health and various pathologies; for example, to examine the effects of exercise (Kvernmo et al., 1998), acute hyperglycemia (Silva et al., 2021), thermal testing^5^ (Mallette et al., 2017), or massage (Rodrigues et al., 2020) on the regulation of microcirculation in healthy subjects, as well as the effects of obesity (Andreieva et al., 2021), DM1 and DM2 (Sun et al., 2012; Mizeva et al., 2018), acute myocardial infarction (AMI) (Ažman-Juvan et al., 2008), AH (Rossi et al., 2011), or peripheral arterial obstructive disease (Rossi et al., 2005a; Rossi et al., 2005b) on possible microcirculatory dysfunction in patients. The main results of some of these studies are summarized in Tables 1, 2 and are discussed in more detail in the following subheadings, with emphasis on the respective frequency bands. The subsequent frequency bands are presented by decreasing frequency rather than in order of physiological importance.

### 3.1 Cardiac influence

Microcirculation delivers nutrients to cells, removes waste metabolites, and eliminates excessive heat. The cellular needs of an individual vary and are influenced by a variety of factors, such as activity level and outdoor temperature^6^ (Ticcinelli et al., 2017). The cardiovascular system must thus adjust its response, i.e., the heart rate, stroke volume, and therefore, cardiac output, according to individual’s current needs. Using WA of the LD signal, it is possible to evaluate the contribution of the mechanisms related to the cardiovascular system to overall microcirculatory control by observing the corresponding frequency band related to cardiac action (0.6–2 Hz).

So far, WA has revealed that the cardiac frequency band is altered in healthy individuals exposed to passive heat stress (increased power of the cardiac frequency band) (Mallette et al., 2017) or lower limb massage (increased activity of the cardiac frequency band) (Rodrigues et al., 2020), as well as in obese patients (increased maximum amplitude of the cardiac frequency band) (Andreieva et al., 2021), and those who had suffered AMI (decreased mean amplitude of the cardiac frequency band) (Ažman-Juvan et al., 2008). Obviously, different studies used different parameters to evaluate WA, rendering direct comparison and conclusions questionable.

It is interesting to note that simultaneous increase in the cardiac, respiratory, and neurogenic frequency bands was observed in a study investigating the effect of passive heat stress on the microcirculatory response in healthy individuals (Mallette et al., 2017). According to the authors, the increased power in the cardiac frequency interval likely reflects an increase in heart rate to compensate for the drop in peripheral resistance caused by neurogenic-mediated cutaneous vasodilation. Thereby, these findings demonstrate that the cardiac, respiratory, and neurogenic systems act in concert to respond to microcirculatory challenges, which makes precise discrimination of a particular system a difficult task.

Furthermore, it is well known that obesity is an independent cardiovascular disease risk factor and one of the major causes of increased risk for other diseases, including insulin resistance, AH, and atherosclerosis (Cercato and Fonseca, 2019). The results of a study conducted on obese patients without other underlying cardiovascular pathology revealed that cardiac influence predominated among centrally mediated mechanisms of microcirculation regulation, with the increase in maximum amplitude of the cardiac frequency band increasing with an increase in BMI (Andreieva et al., 2021). This finding may again reflect the activity of the heart pump trying to compensate for a decline in the vessel’s tone, which the authors speculate may have developed from local spasms occurring in obese patients.

### 3.2 Respiratory influence

The frequency interval between 0.145 and 0.6 Hz is associated with respiration-dependent oscillations caused by intrathoracic pressure changes, influencing venous return to the heart and consequently cardiac output and arterial pressure (Mizeva et al., 2021). During spontaneous inspiration, venous return increases as pressure in the thoracic cavity decreases: the thoracic vena cava dilates, resulting in decreased stroke volume, decreased cardiac output, and consequently decreased arterial pressure; after which, the drop in arterial pressure is detected by the arterial baroreceptors, resulting in a reflex increase in heart rate, with the opposite being true for spontaneous exhalation (Caroll, 2007; Meredith et al., 2012).

Previous studies have investigated respiratory system–related blood-flow oscillations in the microcirculatory system and their interaction with the cardiovascular system using various tests (Tankanag et al., 2020). Considering the results of the studies employing WA, they revealed a significant increase in the respiratory component when applying passive heat stress (increased power of the respiratory frequency band) (Mallette et al., 2017), massage (increased activity of the respiratory frequency band) (Rodrigues et al., 2020), or general anesthesia (increased relative amplitude of the respiratory frequency band) to healthy individuals (Landsverk et al., 2007). Conversely, a significant decrease in the mean amplitude of the band associated with respiratory influence was observed in a study performed in patients who had suffered AMI (Ažman-Juvan et al., 2008).

One of the aforementioned studies exposed healthy individuals to passive heating (Mallette et al., 2017). This resulted in a simultaneous increase in respiratory and cardiac frequency band power, which may be related to cardiovascular adaptation to heat stress (Rowell, 1974). Furthermore, the observed increase in respiratory frequency band power may also be related to hyperthermia-induced hyperventilation, which had previously been shown to modulate the respiratory frequency band amplitude (Estañol et al., 2008), but pursuant to respiratory rate change not being measured in this study, the latter could not be confirmed.

Another recent study used WA to potentially gain mechanistic insight into the effects of effleurage massage (Rodrigues et al., 2020), and the positive contribution of this to individual’s wellbeing is described elsewhere (Vickers and Zollman, 1999). Effleurage massage was applied both proximally (upward) and distally (downward). LD signals were measured in the massaged and non-massaged contralateral limb, and the measurements were divided into three phases: baseline, massage, and recovery. LD signal analysis revealed that the activities of the cardiac and respiratory bands were significantly larger during the massage phase than during the baseline phase when applying both procedures, and significantly larger during the recovery phase than during the baseline phase when applying the proximal procedure. The observed increases have been related to increased local perfusion noticed in both limbs (Rodrigues et al., 2020). Because these observations agree with other measurements performed in this study, most notably a decrease in blood pressure observed during the massage phase, it can be concluded that cardiac and respiratory physiological influences play a crucial role in reestablishing microcirculatory homeostasis after applying effleurage massage.

Finally, a study investigating the effect of general anesthesia on microcirculatory reactivity revealed that the only significantly increased frequency band was the one associated with respiratory influence (Landsverk et al., 2007). These findings are supported by other studies using various approaches and investigating the effects of general anesthesia, in both spontaneously breathing patients and patients exposed to mechanical ventilation, revealing that anesthesia affects the respiratory system and breathing patterns (De Backer et al., 2009; Hedenstierna and Edmark, 2015). It should however be taken into account that the influence may also depend on the type and dose of the anesthetic used (Longnecker, 1984).

### 3.3 Myogenic influence

The frequency range associated with myogenic oscillations correspond with the band from 0.052 to 0.145 Hz. These oscillations, which are triggered by variations in luminal pressure, regulate the internal diameter of the arteries and arterioles, independent from other influences. They can cause either vasodilatation, which lowers vascular resistance and increases blood flow, or vasoconstriction, exerting an opposite effect (Jackson, 2021).

The classic myogenic response to an increase in luminal pressure was originally described by Bayliss (Bayliss, 1902). In addition, previous studies indirectly confirmed these oscillations by excluding the effect of the sympathetic nervous system and respiration (Landsverk et al., 2007). However, the precise molecular mechanisms by which vSMC detect pressure changes remain to be elucidated. As described in a recent study (Jackson, 2021), it is unclear whether the primary variable sensed in the vessel wall is wall strain or wall stress. Furthermore, various mechanosensitive sensors have been proposed, including G-protein–coupled receptors, cation channels, epidermal growth factor receptors, membrane-bound tumor necrosis factor (mTNF*α*), the TNF*α* receptor, and downstream sphingosine-1-phosphate (S1P) signaling. In addition, it is unclear whether these elements act independently or in concert, or whether their expression varies between different vessels and different pathological conditions.

Regardless of the underlying sensory mechanisms, it is generally accepted that their activation leads to depolarization and activation of voltage-gated calcium channels, which leads to an increase in intracellular calcium ions and, ultimately, to vSMC contraction or relaxation (Jackson, 2020), depending on the ratio of myosin light chain kinase and myosin light chain phosphatase activity.

Most studies applying thermal tests and using WA as an analytical tool have revealed that the myogenic frequency band was altered (Table 1). An increase in power and spectral energy was observed in healthy individuals exposed to heating protocols (Mallette et al., 2017; Mizeva et al., 2018). As the myogenic frequency band is associated with the rhythmic oscillations of the pre-capillary sphincter (Mizeva et al., 2018), the observed increases may indicate that hyperthermia causes stretching of the pre-capillary arterioles, affecting myogenic oscillations.

Interestingly, the same study (Mizeva et al., 2018) also investigated the impact of locally applied cooling (to 25°C) and found no significant variations in the spectral energy of the myogenic frequency band. Instead, small variations in the neurogenic frequency band were observed, reflecting sympathetic nervous system stimulation. These results thus demonstrate that heating and cooling activate different physiological mechanisms, which could be speculated because these two provocations result in opposing effects (i.e., vasodilation and vasoconstriction, respectively). However, because cooling tests are used far less than heating tests, more research is required to more accurately define the mechanisms engaged during cooling.

Contrary to the results obtained by applying thermal tests on healthy individuals, studies examining the effect of heating in DM patients did not confirm a consistent increase or decrease in the myogenic frequency band. For example, the results of a study investigating SkBF oscillations in patients with DM2 and with a history of foot ulcers revealed that applying local heating up to 42°C resulted in a decreased normalized amplitude in the myogenic, neurogenic, and endothelial frequency bands (Jan et al., 2013). The observed decrease in myogenic activity agrees with the results of a study using WA of skin temperature oscillations in DM2 patients, but it disagrees with the results of a study performing thermal tests in both DM1 and DM2 patients (Table 1) (Podtaev et al., 2015; Mizeva et al., 2018). In addition, statistically significant differences in this oscillatory component were also not confirmed by another study applying local heating (up to 43°C) and using WA of the LD signal in patients with DM1 (Sorelli et al., 2019). Thus, these results suggest that DM2 likely alters the intrinsic myogenic activity of the vSMC, whereas DM1 presumably does not. However, due to discrepancies in the results of different studies, firm conclusions cannot be drawn.

### 3.4 Neurogenic influence

Neural mechanisms are among the most important mechanisms controlling the response of SkBF to various thermoregulatory and non-thermoregulatory challenges^7^ (Lenasi, 2011). Previous studies using local and ganglion nerve blocks revealed that the neurogenic contribution to microvascular regulation correlates with oscillations whose frequency ranges from 0.021 to 0.052 Hz (Landsverk et al., 2007; Bagno and Martini, 2015). Due to the complex physiological background of this influence, however, great caution is required when interpreting the analysis results. The sympathetic noradrenergic vasoconstrictor fibers, which release both noradrenaline, acting on post-synaptic *α*_1_ and *α*_2_-adrenergic receptors, and various cotransmitters (including neuropeptide Y) (Charkoudian, 2010), are primarily activated during exposure to cold, to reduce SkBF and thus conserve heat and maintain body temperature (Kellogg et al., 1998). On the other hand, the sympathetic active vasodilatory nerve fibers are activated when body temperature increases, such as during exposure to thermal stress or exercise, demanding an increase in SkBF (Lenasi, 2014). It has been estimated that active vasodilator system is responsible for 80%–95% of the SkBF elevation which accompanies heat stress (Kellogg et al., 1998). It has also been hypothesized that the sympathetic active vasodilator system includes a co-transmission mechanism; however, due to the system’s complexity, the exact co-transmitter remains unknown (Wong and Hollowed, 2016). It also remains unclear as to whether NO is involved in cutaneous active vasodilation in humans during hyperthermia (Kellogg et al., 1998).

Accordingly, the results of studies using WA, presented in Table 1, clearly show alteration of the neurogenic component when using thermal tests. A study that examined the effect of passive heating in healthy volunteers revealed increased power in the neurogenic frequency band, probably reflecting cutaneous reflex vasodilatation (Mallette et al., 2017). The same study (Mallette et al., 2017) also found increased power in the endothelial NO-independent band, but not in the endothelial NO-dependent band. Altogether, these results confirm that neurogenic and endothelial NO-independent mechanisms mediate the cutaneous reflex vasodilatation caused by passive heat stress, an idea previously outlined in various studies (Charkoudian, 2010; Kellogg et al., 2012; Johnson et al., 2014), and they further support the idea that in human microcirculation the NO-independent endothelium-mediated mechanisms of vasodilation are more important than the NO-dependent ones (Vanhoutte, 1989; Lenasi, 2011).

In addition, Mizeva et al. (2018) and Jan et al. (2013) found decreased power and spectral energy of the neurogenic component in patients with DM1 and DM2, implying that neurogenic control of microcirculation is impaired in both types of diabetics. Similarly, a decreased spectral amplitude of the frequency band associated with nervous system activity was also confirmed by studies using the FT approach to analyze skin blood flowmotion in diabetics (Bernardi et al., 1997; Lefrandt et al., 2003). Considering that a hyperglycemic state characteristic of diabetes is known to induce neuronal injury to both the parasympathetic and sympathetic nervous systems through different pathways (Yu and Lee, 2021), these results seem expected.

Another study revealing alternations in the neurogenic frequency band was conducted on patients who suffered an uncomplicated AMI (Ažman-Juvan et al., 2008). Because previous studies demonstrated that AMI causes both the activation of the sympathetic nervous system and the release of vasoactive substances (Karlsberg et al., 1979; McAlpine et al., 1988), the authors sought to investigate how the presumed neurohumoral activation affected SkBF and its frequency components. Unfortunately, the results revealed decreased mean amplitudes of all oscillatory components, which made identification of the dominant mechanism regulating SkBF redistribution after AMI impossible. However, the authors speculate that these findings, together with the observed decrease in SkBF, probably reflect the aforementioned neurohumoral activation, which they believe acts as a compensatory mechanism to maintain pressure and flow to vital organs despite decreased cardiac output caused by AMI.

WA was also used previously to evaluate the effects of AH on microcirculatory control in hypertensive patients (Rossi et al., 2011), revealing alternations in the amplitudes of the neurogenic frequency band, which the authors relate to sympathetic overactivity previously associated with AH (Grassi, 2010). Interestingly, the effects of hypertension on the neurogenic frequency band were also demonstrated by using the FFT algorithm in a study by Gryglewska et al. (2010) but not in a study by Rossi et al. (2006a). In addition, Rossi et al. (2006a) found a significant increase in the amplitude of the endothelial frequency band in AH patients, which was also detected in the WA-based study by the same author (Rossi et al., 2011) but not in a study by Gryglewska et al. (2010). Thus, it can be concluded that the WA and FT studies dealing with AH differ in results. However, since there are some discrepancies in the results of the studies using the same analysis technique (FT, (Rossi et al., 2006a; Gryglewska et al., 2010)), we cannot claim that the observed differences are primarily due to the analysis technique used.

### 3.5 Endothelial influence

Endothelial influence refers primarily to the ability of the vascular endothelium to release vasodilatory substances in response to physical forces (shear stress) and to neural and humoral mediators. These substances directly relax the underlying vSMC *via* NO-dependent or NO-independent mechanisms (most importantly, prostacyclin, or PGI_2_, and other prostanoids, and the endothelium-derived hyperpolarizing factor, or EDHF) (Lenasi, 2011; Lenasi, 2016). Additionally, previous studies using vasodilator synthesis pathway inhibitors suggest probable crosstalks between various mechanisms (Lenasi, 2008; Lenasi and Strucl, 2008); however, these interactions are not yet well defined in human skin microcirculation.

Because impairment of endothelial function is linked to various cardiovascular diseases, its role is often assessed in clinical practice; for example, by the flow-mediated dilation (FMD) method (Gori et al., 2012; Cercato and Fonseca, 2019). However, this method allows evaluation of larger conductance rather than microvessels.

The frequency interval corresponding to endothelial influence ranges from 0.005 to 0.021 Hz. To distinguish between the influence of NO-dependent and NO-independent mechanisms, this frequency interval is occasionally separated into two frequency bands: endothelial NO-dependent (0.0095–0.021 Hz), and endothelial NO-independent (0.005–0.0095 Hz) (Silva et al., 2021).

Even though the crucial role played by the vascular endothelium in regulating microcirculation has been confirmed in various animal and human studies (Vanhoutte, 1989), studies using WA to assess endothelial function are scarce. However, the findings presented in Table 1 show that WA is a promising tool for assessing endothelial function in both healthy and diseased individuals. In the first place, WA revealed that the endothelial function is affected by general anesthesia (Landsverk et al., 2007) and passive heat stress (Mallette et al., 2017) in healthy individuals. In addition, WA was successfully employed to study endothelial dysfunction manifested in various disorders, such as obesity (Andreieva et al., 2021), DM (Mizeva et al., 2018) or AMI (Ažman-Juvan et al., 2008).

To start with, DM is known to cause microvascular dysfunction by impairing endothelium-dependent vasodilation, vasomotion, and neurogenic regulation (Silva et al., 2021; Mizeva et al., 2018; Jan et al., 2013). Studies by Jan et al. (2013) and Mizeva et al. (2018) revealed that imposing thermal stress in patients with DM1 and DM2 leads to impaired microvascular reactivity. Specifically, impaired endothelial function, characterized by decreased normalized amplitude of the endothelial frequency band, was observed in DM2 patients with peripheral neuropathy exposed to a heating protocol designed to induce a biphasic vasodilatory response (Jan et al., 2013). In this response, the first peak is believed to correspond to axon–reflex mediated vasodilation (around 35°C), arising from the activation of sensory peptidergic nerve fibers, and the second peak to NO-mediated vasodilation (around 42°C) (Strain and Paldánius, 2018). Accordingly, the results revealed decreased normalized amplitude of the neurogenic frequency band during the first vasodilation peak, and decreased normalized amplitude of the endothelial frequency band during the second vasodilation peak. Endothelial impairment may reflect decreased NO production, partly resulting from increased oxidative stress, and increased endothelial vasoconstrictor production, both of which are typical for DM (Strain and Paldánius, 2018). Nevertheless, as emphasized by the authors, age and BMI differences between diabetics and healthy controls may have influenced these results, especially because studies show that age and BMI may affect the mechanisms underlying microcirculatory control (Hodges et al., 2017; Andreieva et al., 2021).

While the negative role of DM-related long-term hyperglycemia on microcirculation regulation is well known, the impact of acutely elevated blood glucose levels remains far less understood. The findings of current studies in this field are scarce and mostly contradictory. For example, whereas Houben et al. (1996) found that acute hyperglycemia does not affect endothelium-dependent or -independent vasoreactivity, a recent study by Loader et al. (2017) revealed that it impacts pathways affecting NO bioavailability, consequently causing decreased endothelial function and decreased vasodilation capacity in healthy individuals. However, it should be mentioned that the methods used in these studies differ. The authors used various imaging techniques and assessed skin microcirculation after various times from a hyperglycemic load, which makes a direct comparison of the results questionable.

Finally, the presumed opposing effects of acute hyperglycemia and hyperinsulinemia on the endothelium should also be mentioned. It is well established that increased levels of insulin associated with hyperglycemia affect endothelium signaling pathways, causing either vasodilation through increased NO production or vasoconstriction through increased endothelin production (Rajapakse et al., 2013). In addition, spectral analysis of LD signals based on FT confirmed that skin microvascular function is influenced by the metabolic and vasodilatory effects of insulin (Serné et al., 2001; Rossi et al., 2005a; Rossi et al., 2005b), and that systemic hyperinsulinemia in skin affects endothelial NO-dependent vasodilation as well as vasomotion (Serné et al., 2002). However, how the effects of hyperglycemia and hyperinsulinemia integrate at the level of the microcirculation remains to be elucidated.

A study conducted by Silva et al. (2021) was the first to use WA to investigate the effect of acute oral glucose load (OGL) on the spectral components of cutaneous microcirculation LD signals. The signals were acquired from the finger pulp and the volar forearm of healthy volunteers. Results revealed that a standard OGL induces site-specific short-term effects on cutaneous microcirculation, with elevated blood glucose levels persisting for at least 60 min after glucose indigestion, which agrees with the results reported by Loader et al. (2017). Because an increase in blood glucose concentration is followed by relatively rapid secretion of insulin, which is associated with NO-dependent vasodilation, a change in endothelial NO-dependent frequency band would be expected. The WA results, however, revealed that acute hyperglycemia did not significantly affect the activity of the endothelial NO-dependent frequency band. Previously mentioned opposing actions of glucose and insulin could be taken into account. To further study possible crosstalks, tracing plasma insulin concentration may be advisable in future studies.

While glucose load had little overall effect on the endothelial NO-dependent component, the endothelial NO-independent component could not be evaluated due to the time series being too short. Results, however, suggest that the observed microcirculatory effects of acute hyperglycemia probably involve modulation of the sympathetic nervous system activity. Due to large disparities in the results of various studies and the complex interplay of physiological mechanisms underlying the microcirculatory response to acute hyperglycemia, further research and analytical improvements in this field are required.

## 4 Limitations and future perspectives

WA of the LDF signal might be regarded as a promising tool to assess microcirculatory dynamics offering advantages over methods such as FFT. The WA-assisted signal processing may enable the extraction of additional physiological parameters for clinical diagnosis. Nevertheless, some limitations and unresolved problems of the WA approach must be highlighted, especially because different physiological activities may have different effects on the microcirculatory signals.

The exact physiological mechanisms underlying the observed oscillations in skin microcirculation, especially of those lower than 0.1 Hz, are not completely elucidated (Ticcinelli et al., 2017). Accordingly, the arbitrary intervals of the physiological frequency bands discussed above are not fixed and vary from author to author (Stefanovska et al., 1999; Bagno and Martini, 2015; Reynès et al., 2020; Andreieva et al., 2021; Silva et al., 2021). Therefore, interpretation of observed frequencies, especially those belonging to borderline areas, depends on the selection of the frequency intervals, which hampers direct comparisons among studies. In addition, various factors can cause a slight shift in the boundaries of the frequency bands. For example, temperature and emotional reactions are known to influence oscillations of neurogenic origin (Landsverk et al., 2007). As a result, the differing experimental conditions utilized may to some extent explain why studies have evaluated the role played by sympathetic nervous system activity in microcirculatory control differently.

Therefore, one should always consider the complex interplay of the various biological, environmental, and physiological factors involved in skin microcirculation when evaluating microcirculatory signals (Lenasi, 2014). So far, there is no uniform technique to correctly evaluate the impact of single factors on skin microcirculatory response. To this end, the approach discussed in this article, aiming to divide the LD microcirculatory signal into precisely defined physiological frequency bands, each related to one physiological influence only, is rather robust and debatable, and performing additional tests is advisable to obtain the clearest possible results. Moreover, data should be interpreted in accord with data obtained from other valuable biological signals (e.g., heart rate variability to evaluate autonomic nervous system activity) as well as with the clinical status of the patient.

When analyzing blood-flow signals, the problem of edge effects may affect the results at large scales because applying WT to finite-length time series engenders time lags (Reynès et al., 2020). These time lags result from the center of the wavelet function being shifted from both ends of the finite-length series by a value equal to half the length of the analyzing wavelet, which results in missing values and consequently abnormally large amplitudes at the end of signals. To avoid time-lag effects, long LD signals must be recorded; however, increasing recording time gives rise to motion artifacts, imposing a further limitation on spectral analysis. Some authors suggest utilizing adaptive-WT to resolve this problem, that is, concurrently reducing recording time and obtaining reliable results, but this technique is not yet widely used in practice (Tankanag and Chemeris, 2008).

No clear or unique guidelines for LDF recording time exist in the literature. The Nyquist-Shannon sampling theorem states that the lowest detectable frequency is inversely proportional to the signal duration (Bernjak and Stefanovska, 2007), which means that, for example, an LDF recording of 105 s should be sufficient to detect oscillations with a frequency of about 0.0095 Hz. In practice, the length of LD signal recordings varies considerably (Table 1). The appropriate recording duration is determined by a variety of factors, including the anatomical site of interest, the study population, and the frequency band of interest. Considering all mentioned, the traditional recommendation is that at least 20 min of signal recording is required to provide quality resolution of the low-frequency intervals (Stefanovska et al., 1999; Hodges et al., 2017; Reynès et al., 2020). On the other hand, a recent study shortened 20 min of basal LD measurements recorded at two different anatomical sites (Reynès et al., 2020). The signal was shortened every minute until the last 10 min. WA results of the shortened recordings were compared to the WA result of the 20-min reference recording. The study’s main finding was that LDF recordings should last at least 15 min on the forearm and at least 13 min on the dorsum of the foot to provide a reliable WA assessment of the three lowest frequency bands. However, because this study was conducted on healthy participants, these recommendations should be further validated in patients.

At this point, it is worth noting that the Nyquist-Shannon sampling theorem determines not only the lowest detectable frequency, but also the highest. Unlike the lowest detectable frequency, which is determined by the duration of the signal, the highest detectable frequency is defined as the half-value of the signal’s sampling frequency (Bernjak and Stefanovska, 2007). In practice, various sampling rates are used, but given the range of frequency intervals important for microcirculation assessment (0.005–2 Hz), they are generally much higher than the minimum required by the Nyquist-Shannon sampling theorem (for example, Rossi et al. (2011); Jan et al. (2013); Rodrigues et al. (2020); Reynès et al. (2020) all used 32 Hz; Landsverk et al. (2007) used 40 Hz; Silva et al. (2021) used 500 Hz).

Another problem that has long been acknowledged is quite large spatial and temporal variability of the LDF signals measured using a standard probe. The LD signals measured, and their spectral components, vary greatly with the measurement location (Hodges and Del Pozzi, 2014; Silva et al., 2021) partly due to the spatial anatomic heterogeneity of the distribution of microvessels in the dermis (Podtaev et al., 2015), and partly due to the construction of the LDF, which covers only around 1 mm^2^ of the skin area^8^ (Ažman-Juvan et al., 2008).

Variations in the LD signal measured between repeated measurements have also often been reported (Lenasi, 2014; Tamas et al., 2016), which may result in poor reproducibility of the results. Increasing the recording area (e.g., by using LD imaging or LSCI instead of LDF) is one way to improve signal reproducibility, but it should be noted that time-frequency data analysis is much more challenging in this case (Podtaev et al., 2015), and the imaging technique has also been shown to have its limitations (Briers et al., 2013).

Finally, it should be mentioned that no consensus exists on the WA parameter that would be most suitable for describing the properties of the WA-reconstructed LD signal spectra. Various absolute or relative parameters are evaluated in practice (Table 1; Section 2.3). Authors frequently base their conclusions on calculations of absolute or maximum amplitude at the frequency interval of interest. Considering that the exact amplitudes of peaks change with time (Figure 1A) as well as that the exact positions of peaks differ between subjects (Stefanovska et al., 1999), we believe that future research should prioritize the use of relative measures, especially those relying on calculations of spectral power or activity, which are currently used by a small number of researchers. Doing so would likely reduce the inter-subject variability that is typical of LD signals and consequently their related WA spectra (Silva et al., 2021).

### 4.1 Potential applications of WA

Ideally, the high-quality LD signals could allow quantitative comparison with other microcirculatory signals, which would help clarify the difference between physiological factors in their effect on microcirculatory signals. For example, PPG, which reflects skin blood volume pulsation (Bhoi and Sarkar, 2012), is sometimes thought of as a low-cost replacement for LDF (Rodrigues et al., 2019). Studies on the use of PPG show that respiratory rate significantly affects the baseline, amplitude, and frequency of PPG signals (Liu et al., 2020), whereas blood pressure (Khalid et al., 2020) and anesthesia (Khalid et al., 2022) affect the waveform features of PPG signals. Because some studies have also found a partially good correlation between PPG- and LDF-measured signals (Mizeva et al., 2015; Silva et al., 2016), these results may be helpful in differentiating between physiological factors affecting microcirculation. Nevertheless, caution is needed when directly comparing LDF and PPG results, especially because these two techniques use different light wavelengths despite their similar biophysical basis (Bhoi and Sarkar, 2012; Fedorovich et al., 2014; Rodrigues et al., 2019). This means that they have different skin penetration capabilities (for example, LDF, which uses a wavelength of 780 nm, provides a penetration depth of about 0.3–0.5 mm, while PPG, which uses a wavelength of 530 nm, provides a perfusion measurement 2 mm deep (Rodrigues et al., 2020)) and thus different discrimination capabilities. Thus, they reflect the activities of the different skin layers, which are characterized by different anatomical structures (Rodrigues et al., 2020). Finally, not many studies have evaluated perfusion using both methods (Silva et al., 2016). Those that have done so have found not only correlations between the frequency bands, but also discrepancies (Rodrigues et al., 2019; Rodrigues et al., 2020). Therefore, much remains to be clarified before the exact value of the combined application of these two techniques can be determined.

As WA of LDF-measured microcirculatory signals potentially offers insight into physiological influences that regulate the microcirculation, application of this approach in the clinic could allow timely detection of subtle alternations known to predispose to microvascular dysfunction in various cardiovascular and metabolic diseases. For example, DM, AH, peripheral arterial obstructive disease, and atherosclerosis are known to cause abnormalities of skin microcirculation by impairing various physiological influences. DM and peripheral arterial obstructive disease have been associated with endothelial dysfunction, altered vasomotion, and neurogenic regulation (Rossi et al., 2006b; Mallette et al., 2017; Silva et al., 2021; Jan et al., 2013); factors characterizing AH presumably include impaired endothelial function and altered neurogenic regulation of vascular tone (Westfall, 2006; Konukoglu and Uzun, 2017; Grassi, 2021; Xu et al., 2021), and the development of atherosclerosis has been associated with underlying endothelial dysfunction (Sitia et al., 2010; Xu et al., 2021). In this context, WA-based spectral analysis of LD microcirculatory signals may provide a good starting point for defining new biomarkers, potentially by defining characteristic WA spectra for a disease of interest. However, because of the limitations discussed previously, including wide discrepancies in the results of different studies and disputes about the exact physiological mechanisms underlying the observed oscillations, further standardization of the WA approach is needed before potential WA-based biomarkers can be translated into routine clinical practice.

## 5 Software application

Considering the studies discussed in this article, most authors used MATLAB (The Mathworks Inc., Natick, MA, United States of America) to perform WA (Landsverk et al., 2007; Ažman-Juvan et al., 2008; Rossi et al., 2011; Mallette et al., 2017; Jan et al., 2013; Rodrigues et al., 2020; Silva et al., 2021), whereas others used Mathematica (Wolfram, Hanborough, United Kingdom) (Mizeva et al., 2018) or did not specify which software was used to perform WA (Andreieva et al., 2021). When using these approaches, it is important to correctly specify the parameters of the raw signal, such as sampling frequency, period, number of sampling points, and so on. The properties of the desired spectrum should also be specified, including the type of spectrum (for example, three-dimensional wavelet spectrum, time-averaged wavelet spectrum, etc.), the WA parameters to be computed, and the boundaries of the frequency bands. The family of wavelet functions and their parameters, in particular the symmetry and time width parameters (Olhede and Walden, 2002; Lilly and Olhede, 2010), which affect the shape of the wavelet and, consequently, the quality of the reconstructed spectra in terms of their temporal and frequency resolution, must also be specified. Finally, we can conclude that while employing custom-written programs to perform WA makes it easy to tailor the computational process to one’s needs, it also inevitably leads to discrepancies in results between research groups. However, because there is currently no standardized tool for performing WA, such as the commonly used software from Moor Instruments (Axminster, Devon, United Kingdom), Nevrokard (Medistar, Slovenia), or Perisoft (Stockholm, Sweden) for the application of FT, we believe that there is a need to develop algorithms and software for similar application of microcirculatory signal-customized WA. Thus, the field undoubtedly offers great opportunities for future research and software development.

## 6 Concluding remarks

To the best of our knowledge, this review represents one of the first attempts to highlight and summarize WA’s key advantages and disadvantages, the resolution of which would undoubtedly result in a more accurate assessment of skin microvascular function, potentially also for clinical use.

Unquestionably, the WA of the LD signal is a valuable tool for assessing microcirculatory dynamics; however, several limitations remain in practice. First, a consensus is needed on the LD recording duration required for a reliable assessment of the three lowest frequency bands. In addition, to facilitate direct comparisons between different studies, efforts should be made to standardize not only the boundaries of the frequency bands associated with different physiological influences but also the use of WA parameters to describe the contributions of the different frequency bands to the overall microcirculatory control.

Altogether, these advances would help in quantifying skin microcirculation more accurately, potentially enhancing an early detection and further follow-up of diseases characterized by microvascular dysfunction.

## Footnotes

1 Skin microcirculation is convenient for measurement as it is easily accessible, making it an appropriate model for assessing generalized microvascular function (Lenasi, 2011). Furthermore, because microvascular dysfunction is a systemic process encompassing similar mechanisms throughout the body, it is reasonable to assume that impairment of skin microcirculation might reveal generalized microvascular dysfunction and as such be a hallmark thereof (Treu et al., 2017).

2 In LDF, the incident laser beam interacts with moving particles in tissues, mostly erythrocytes, which causes a wavelength shift proportional to the number and velocity of the moving erythrocytes. The amplitude of the LD signal is proportional to the blood flow and is expressed in arbitrary (perfusion) units (Martini and Bagno, 2018).

3 In the literature, two types of WT and corresponding mother wavelets have been described: continuous and discrete (Delvecchio, 2012). This article concentrates on the continuous WT, which is suitable for analyzing LD microcirculatory signals.

4 PPG is another non-invasive optical imaging technique sometimes used to assess microcirculation (Bhoi and Sarkar, 2012). Yet, it widely outrages the LDF when considering its disadvantages in microcirculation evaluation.

5 Thermal tests usually involve local heating of the skin to a specific temperature to elicit maximal vasodilation (Mizeva et al., 2018).

6 SkBF amounts to approximately 0.3 L/min at rest in thermoneutral conditions. During intensive exercise in environments of increased temperature, it can increase up to 8 L/min, demanding appropriate adaptation of the mechanisms regulating microcirculatory response (Lenasi, 2011; Potočnik and Lenasi, 2016).

7 One should also bear in mind that local substances, especially those released by the vascular endothelium, can significantly modify the response to these systemic challenges (Lenasi, 2011).

8 Depending on the incident laser beam wavelength, these corresponds with a volume of around 1 mm^3^.
